# Acute, Subchronic, and Genetic Toxicity Assessments of a Composition of *Citrus aurantifolia* Fruit Rind and *Theobroma cacao* Seed Extracts

**DOI:** 10.1155/jt/4239607

**Published:** 2024-11-27

**Authors:** Sundararaju Dodda, Sujatha Polavarapu, Krishnaraju Venkata Alluri, Trimurtulu Golakoti, Krishanu Sengupta

**Affiliations:** Department of Toxicology, Laila Nutraceuticals R&D Center, Vijayawada, Andhra Pradesh, India

**Keywords:** comprehensive safety, genetic toxicity, LN19183, no observed adverse effect level, repeated dose 90-day oral toxicity study

## Abstract

LN19183 is a standardized composition of *Citrus aurantifolia* (Christm) Swingle (CA) fruit rind and *Theobroma cacao* L. (TC) seed extracts that have recently been demonstrated to increase resting energy expenditure (REE) and reduce body fat in rats. CA and TC are important herbs in traditional medicine for various health benefits. The present study evaluates the comprehensive toxicity of LN19183 in acute, subchronic, and genetic toxicity studies following the guidelines of the Organization for Economic Co-operation and Development (OECD) for testing chemicals. The acute oral and dermal and 90-day subchronic oral toxicities were performed in rats, and acute dermal and eye irritations were performed in rabbits. In the subchronic toxicity study with a 28-day recovery period, male and female Sprague Dawley rats were orally gavaged with daily LN19183 doses of 500, 1000, or 2000 mg/kg body weight (BW). Furthermore, the genetic toxicity studies included mutagenicity in bacteria, chromosome aberration, and micronucleus assays in human blood mononuclear cells in vitro and micronucleus assay in Swiss albino mice bone marrow in vivo. Acute and subchronic repeat dose oral toxicity studies showed no adverse events, clinical signs, or mortality. All animals exhibited normal food and water intake and natural BW gain. In the 90-day study, LN19183 did not induce major changes in hematology, biochemical evaluations, and urine analysis; gross and histopathological findings did not show any treatment-related lesions or abnormality. The no observed adverse effect level (NOAEL) of LN19183 supplementation was 2000 mg/kg BW/day. In the genetic toxicity studies, LN19183 treatment did not show significant increases in the revertant bacterial colonies, chromosomal aberrations, or number of micronucleated cells. The present observations affirm that oral consumption of LN19183 is safe, and this botanical composition is nonmutagenic and nonclastogenic.

## 1. Introduction

LN19183 is a standardized, proprietary blend of *Citrus aurantifolia* (Christm) Swingle fruit peel or rind and *Theobroma cacao* L. seed or bean extracts. In a recently published study, we demonstrated that LN19183 synergistically increased fibroblast growth factor-21 (FGF-21) production in mouse fat cells in vitro. Also, LN19183 reduced body fat mass and increased resting energy expenditure (REE) in high-fat diet–induced obese rats [1].

*C*. *aurantifolia*, or lemon fruit and rind, is widely used as a cooking ingredient and in beverage and food industries as a flavoring agent or salad dressings worldwide. *C. aurantifolia* fruit peel is rich in bioactive phytochemicals [2]. Pharmacologically active phytochemicals in *C. aurantifolia* extracts, including hesperidin, naringenin, apigenin, and kaempferol, contribute to liver protection and anticancer and antioxidant activities [2–5]. The oil fraction of fruit rinds is used for treating obesity, asthma, arthritis, bronchitis, cold, and sore throat [6]. The essential oil produced as a by-product from citrus processing at the industrial scale has become a new functional ingredient in food preservation [7]. It is recognized as a safe botanical ingredient by the US Food and Drug Administration (USFDA) [8]. *Theobroma cacao* L. beans are used in cocoa beverages and chocolate and confectionery industries. Cocoa bean extracts are rich in phenolic acids, flavonoids, simple phenols, and benzoquinones. Polyphenolic compounds are reported to contribute to the antioxidant potential and provide metabolic and cardiovascular benefits and anti-inflammation [9, 10].

Although the plant raw materials of LN19183 are widely used in food and beverage industries [2, 7, 11], a comprehensive toxicological evaluation of the final composition is vital for safety in human trials. To this objective, we conducted a series of toxicological assessments following the guidelines of the Organization for Economic Co-operation and Development (OECD).

The present study demonstrates the systemic safety of LN19183, conducted in acute and subchronic toxicity studies in animal models. Further, the genotoxicity tests in bacteria and mammalian cells establish that LN19183 is not mutagenic or clastogenic.

## 2. Materials and Methods

### 2.1. Chemicals, Reagents, and Bacterial Strains

The reagents for hematology and serum chemistry assays were sourced from Mindray (Shenzhen, China) and Ilab Aries (Bergamo, Italy), respectively. Bacto Agar and the laboratory reagents, including sodium azide, cyclophosphamide, 2-aminoanthracene, 2-nitrofluorene, 9-aminoacridine, 4-nitroquinoline-1-oxide, mitomycin C, and colchicine, were sourced from Becton Dickinson (Sparks, Maryland) and Sigma-Aldrich Chemicals (St. Luis, MO), respectively.

*Salmonella typhimurium* (TA98, TA100, TA1535, and TA1537), *Escherichia coli* (WP2 uvrA), and the rat liver S9 fraction were sourced from Molecular Toxicology Inc., Boone, NC.

### 2.2. Test Material

LN19183 is a standardized composition containing two parts of aqueous ethanol (50%) extracts of *C. aurantifolia* fruit peel (CA) and one part of water extract of *T. cacao* bean (TC). The plant material collection, extraction procedure, and standardization are described elsewhere [1]. Briefly, the dried plant materials were purchased from local vendors in Andhra Pradesh, India, and verified by a taxonomist, and the specimen CA (LNR6824) and TC (LNR6924) samples are stored at Laila Impex R&D Centre, Vijayawada, India. The dried CA was pulverized, and the coarse powder was extracted with 50% aqueous ethanol at 50°C to 60°C under continuous percolation for 14 to 16 h. The coarse powder of dried TC was extracted with water at 50°C to 55°C for 3 h. The extracts were concentrated to either thick paste or dry powder under vacuum and blended at a 2:1 ratio. The final product, LN19183, contains an 80% herbal blend and 20% excipients (18% glucidex and 2% syloid). LN19183 is a light to dark brown powder, approximately 70% water soluble. The standardized LN19183 contains at least 5.0% citric acid, 0.5% theobromine, and 0.3% hesperidin. Consistent batches of LN19183 were produced in a CGMP-certified manufacturing unit of Laila Nutraceuticals, Vijayawada, India.

### 2.3. Ethics Approval

The Institutional Animal Ethics Committee (IAEC) of Laila Nutraceuticals, Vijayawada, India, approved the study protocols of acute oral (no. LN/IAEC/TOX/LN230602), dermal toxicity (no. LN/IAEC/TOX/LN230603), acute dermal irritation (no. LN/IAEC/TOX/LN181001), eye irritation (no. LN/IAEC/TOX/LN181002), subchronic oral toxicity study (no. LN/IAEC/TOX/LN220308), and in vivo micronucleus assays (no. LN/IAEC/TOX/LN200703). In vitro micronucleus assay and chromosomal aberration assay protocols were approved (no. ECR/563/Inst/AP/2014/RR-20) by the Institutional Ethics Committee of Alluri Sitarama Raju Academy of Medical Sciences (ASRAM) Hospital, Eluru, Andhra Pradesh.

### 2.4. Animals and Husbandry

Animal handling and care procedures were registered (no. 1668/PO/RcBi/S/12/CPCSEA) with the Committee for the Purpose of Control and Supervision of Experiments on Animals (CPCSEA), India. Male and female Sprague Dawley rats (9–12 weeks old) and 3-month-old white New Zealand rabbits (male, BW 2.0–2.4 kg) were procured from Vivo Biotech Limited (Hyderabad, India) and Gentox Bio Services Pvt. Ltd. (Hyderabad, India), respectively. Six to eight weeks old male (BW 22–32 g) and female (BW 18–24 g) Swiss albino mice were purchased from VAB Biosciences (Hyderabad, Telangana, India).

Animals were accustomed to the laboratory environments for 7 days before starting the experiments. Individually ventilated cages (IVC) housed the animals, with food and RO water ad libitum. The animals were maintained at a 12-h light/dark cycle at 21 ± 2°C and 40%–70% relative humidity. The standard pellet food was sourced from SDS Diet (Essex, UK).

### 2.5. Acute Studies

#### 2.5.1. Oral Toxicity

Three female Sprague Dawley (SD) rats were used in the single-dose oral toxicity test [12] following the method described earlier [13]. In the limit test, three female SD rats were subsequently used at an interval of 48 h. Five thousand milligrams per kg BW of LN19183 was suspended in sterile water and orally administered to the fasted rats. After the test substance administration, the rats were examined during the first 4 h, subsequently at 24, 48 h, and then daily for 14 consecutive days. Various parameters were recorded daily: mortality, clinical signs, behavioral changes, distress, BW, food, and water consumption. On Day 15, following CO_2_ euthanasia, the rats' major organs and tissues were examined for any gross pathological changes.

#### 2.5.2. Dermal Toxicity, Dermal Irritation, and Eye Irritation

The dermal toxicity test OECD 402 [14] was conducted on female SD rats, and dermal irritation OECD 404 [15] and eye irritation OECD 405 [16] tests were conducted on healthy young male rabbits. Each test was performed on three animals following the methods described earlier [13]. For the dermal toxicity test, moistened LN19183 was smeared on the skin of the test animals at 2000 mg/kg (limit dose) with the help of porous gauze dressings. The application area (4 × 4 cm.) was prepared by clipping the hair from the scapulae to the wing of the ileum and halfway down the flanks. After 24 h, the patch was removed, and the skin was cleaned using moist cotton. The dermal reaction of toxicity was monitored for the subsequent 14 days.

For the dermal irritation test, 0.5 g moistened LN19183 was applied on shaved skin at the dorsal trunk area using a gauge pad (6 × 6 cm) for four hours. The signs of skin irritation were assessed after 1, 24, 48, and 72 hours. The dermal irritation or corrosion, including signs of skin redness and edema, was monitored [17].

The eye irritation test was conducted on one eye of each animal, and the other eye was untreated and considered as the control. A single dose of LN19183 (equivalent to 70 mg) was inoculated, and after 1 h, the eye was washed with saline. Tramadol 50 mg/mL was used in the study to minimize the distress and pain in the animals. Ocular reactions were evaluated after 1, 24, 48, and 72 hours of instillation. Conjunctival redness, chemosis, and corneal opacity were assessed using an ophthalmoscope [17]. After sacrifice, the vital organs were examined for any gross pathological changes.

### 2.6. Subchronic Toxicity

This 90-day oral toxicity study [18] was performed in 7-8 weeks of SD rats following the methods described earlier [13]. The animals were randomized into four main and two recovery groups. Each main group consisted of 10 male and 10 female rats. Group 1: vehicle control (distilled water, G1); Groups 2, 3, and 4: 500, 1000, and 2000 mg per kg BW of LN19183. The main groups of animals were euthanatized with CO_2_ on Day 91. The control (G1R) or LN19183 high-dose (G4R) reversal group consisted of 10 animals (5 males and 5 females). Following the 90-day supplementation, the animals received only a regular rodent pellet diet during the 28-day reversal period. The daily cage-side monitoring included changes in movement, behavior, fur erection, tear secretion, and any other clinical signs. Their BW and food consumption were measured every week. During the last week, the main groups of rats were observed for behavioral and neurological status as a battery of functional observation or functional observational battery (FOB). Overnight fasted rats were mildly anesthetized with isoflurane, and their retro-orbital blood samples were collected.

The K2-EDTA blood samples were analyzed using a hematology analyzer (BC-5000, Mindray, Shenzhen, China). The serum clinical biochemistry parameters and electrolytes were measured using an automated analyzer (BS-40; Mindray, Shenzhen, China) and an electrolyte analyzer (BA180603, Turbolyte, CPC Diagnostics Pvt. Ltd., Chennai, India), respectively. The hematology, plasma biochemistry, and electrolyte parameters are tabulated in Tables 1 and 2.

Thyroid-stimulating hormone, thyronine or triiodothyronine, and thyroxin were measured using ELISA kits (Krishgen Biosystems, Cerritos, CA). The absorbance was read using an ELISA reader (X-Mark; Bio-Rad Laboratories, Hercules, CA, USA). The estrous cycle was evaluated by examining the vaginal smear of the female rats.

At the end of the supplementation period, the overnight fasted animals were euthanized by carbon dioxide asphyxiation, and a gross examination was performed. The major organs such as the liver, kidneys, heart, lungs, brain, spleen, adrenal glands, testes, epididymis with seminal vesicles (males), ovaries, and uterus with the cervix (females) of the control and LN19183-2000 group animals were collected, weighed, and processed for histopathology examination. Small pieces of each tissue were fixed in 10% neutral buffered formaldehyde for 2 days, then dehydrated in graded alcohol, embedded in paraffin, and cut into 4 μm thick sections using a rotary microtome (Leica Biosystems, Nussloch, Germany). The tissue sections were stained using hematoxylin-eosin and were examined under an Axio Scope light microscope (Carl Zeiss, Munich, Germany).

### 2.7. Bacterial Reverse Mutation Assay

This assay was performed using *Salmonella typhimurium* (TA98, TA100, TA1537, and TA1535) and *Escherichia coli* (WP2 uvrA) strains with or without liver fraction S9 following the OECD 471 guidelines [19] to assess the mutagenicity potential (point mutation) of LN19183 following the methods as described earlier [13]. These bacterial cells in suspension were exposed to different concentrations of LN19183, in triplicates, directly on the agar layer [20]. The treated bacterial cultures were maintained at 37°C for 48–72 h. The positive control bacterial cultures received the strain-specific mutagenic compounds.

### 2.8. In Vitro Chromosome Aberration Assay

This assay was performed in lymphocytes isolated from the peripheral blood of a 23-year-old healthy male [21] following the methods described earlier [13]. The lymphocytes were cultured in RPMI 1640 containing 10% FBS and phytohemagglutinin [22, 23]. The LN19183-treated cells were incubated at 37°C for 4 or 24 h. The 4-h treatment duration experiment was conducted with or without (1%, v/v) S9 activation. The long exposure (24-hour) experiment was conducted without the S9 activation. The control cultures received 1% DMSO, and the positive control cultures were treated with cyclophosphamide monohydrate or mitomycin C. After the treatment, the methanol:glacial acetic acid (3:1, v/v)–fixed cells were stained with 5% Giemsa solution. Approximately three hundred cells were counted for mitotic index (MI) and chromosome aberration frequency in each treatment.

### 2.9. In Vitro Micronucleus Test

This test was conducted following the OECD 487 guidelines [24]. The isolated human blood lymphocytes were treated with LN19183 with or without S9 fraction for 4 or 24 h. The control cultures received 1% DMSO. Treatments with mitomycin C, colchicine, and cyclophosphamide were used as positive controls for 4 and 24-hour treatments without S9 and 4-hour treatment with S9, respectively. Following the treatments, the glacial acetic acid:methanol mixture (1:3 v/v)–fixed cells were stained with Giemsa and examined to evaluate micronuclei in the bi-nucleated cells [25].

### 2.10. In Vivo Micronucleus Assay

This assay was conducted in mouse bone marrow [26]. Healthy Swiss albino mice (aged 6–10 weeks) were randomly allocated into five groups (*n* = 10; 5 males and 5 females). The animals received two oral doses at a gap of 24 h with either LN19183 of 500, 1000, or 2000 mg/kg BW or distilled water as vehicle control. Only the positive control mice received one intraperitoneal (i.p.) dose of cyclophosphamide (40 mg/kg BW), 24 h before sacrifice. The femur bone marrow was collected in fetal bovine serum [27, 28]. The absolute methanol-fixed bone marrow cells were stained with 5% Giemsa. The cells were examined under a light microscope (ECLIPSE E200, Nikon Corp. Tokyo, Japan) and scored as described earlier [13].

### 2.11. Statistical Analysis

GraphPad Prism 8.0.2 (GraphPad Software, La Jolla, CA) was used to analyze the data. A *p* value of < 0.05 was considered statistically significant. One-way ANOVA and Student's *t*-test were conducted for comparison analyses in the main groups and in the reversal groups, respectively.

## 3. Results

### 3.1. Single-Dose Oral and Dermal Toxicity

A single-dose oral (5000 mg/kg BW) administration or dermal (2000 mg/kg BW) application of LN19183 showed no toxicity or mortality. The animals appeared healthy, and their BWs were similar to the control animals. The gross pathological observations showed no abnormalities; the treated animals did not show mortality or behavioral anomalies (Supporting Table S1). These results suggest that the median lethal doses (LD50s) of LN19183 for acute oral and dermal toxicity in female SD rats are greater than 5000 and 2000 mg per kg BW, respectively.

### 3.2. Single-Dose Dermal Irritation and Eye Irritation

The experimental rabbits showed no signs of skin reactions in the single-dose dermal irritation test, suggesting the dermal irritation index in LN19183-treated animals is 0 [29].

LN19183 application did not show signs of eye irritation. The cornea and iris appeared normal in the experimental animals. Based on the harmonized integrated hazard classification system, LN19183 is nonirritant to the skin or eyes [29].

### 3.3. Subchronic Repeated Dose Oral Toxicity

LN19183 consumption for 90 consecutive days showed no toxicity and incidences of death in the SD rats. All tested animals were healthy; they survived till sacrifice. The LN19183-supplemented rats showed no neurobehavioral changes.

The BW gain in LN19183-supplemented rats of both genders was comparable with the untreated control rats (Table 3). The weekly food intake by the experimental rats is presented in Table 4. During the first week of supplementation, the low-dose LN19183–fed female and male rats, and on the 12th week, the high-dose–supplemented main group of female rats consumed significantly more food (*p* < 0.05) than the control rats. LN19183-supplemented reversal male rats consumed substantially less food on the 4th week, and the female reversal rats consumed significantly reduced and more food on the 5th and 16th week of the experiment, respectively (Table 4).

Tables 1 and 2 present the blood parameters and serum biochemistry measures of the experimental rats, respectively. These measures remained within the normal physiological ranges. However, MCV was increased in the high-dose LN19183–treated male rats (Table 1), hematocrit values were increased in the mid-dose and high-dose LN19183–treated female rats, and MCHC was decreased in high-dose group female rats compared to the control rats (Table 1). Although these changes were significant, they were within the normal range and posed no safety concerns. LN19183 supplementation did not alter serum TSH, T3, and T4 concentrations compared to the control rats (Supporting Table S2).

**Figure 1:**
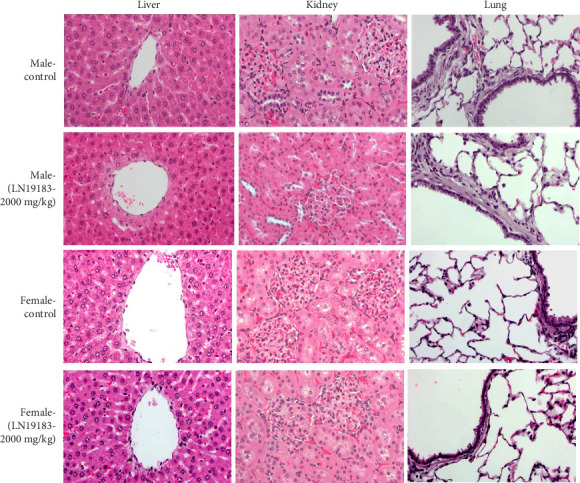
Representative photomicrographs (40x) showing hematoxylin-eosin–stained liver, kidney, and lung tissue sections of control and the high-dose LN19183 (2000 mg/kg per day)–supplemented male and female rats in the 90-day repeated dose oral toxicity study. Microscopic examinations revealed no significant morphological changes in the vital organs of the treatment groups compared to the control animals.

In LN19183-supplemented male and female rats, the vital organs' absolute and relative weights were unaltered (Supporting Tables S3 and S4). Moreover, the pathological examinations revealed no abnormal findings in the experimental rats. The microscopic examinations of the vital organs did not show pathological changes in the LN19183-supplemented rats. Representative photomicrographs of the liver, kidney, and lung tissue sections of the control and high-dose LN19183–supplemented male and female rats are presented in Figure 1.

### 3.4. Bacterial Reverse Mutation Assay

LN19183 treatments up to 5000 μg did not significantly increase the revertant colonies of the tested *E. coli* or *S. typhimurium* strains in the presence or absence of S9 metabolic activation (Table 5). The reference control plates incubated with the strain-specific mutagens significantly increased (*p* < 0.05; one-way ANOVA) the number of revertant colonies. These data indicate that LN19183 treatment did not cause point mutation in the bacterial strains.

### 3.5. In Vitro Micronucleus and Chromosome Aberration Assays

LN19183 treatments up to 1250 μg/mL in human lymphocytes did not induce micronuclei formation (Table 6). Similarly, up to 1250 μg/mL, LN19183-treated lymphocytes did not show structural chromosome aberrations (Supporting Table S5). These observations were consistent in the short and long duration of LN19183-treated cultures. These data indicate that no chromosome break happened due to the LN19183 treatment; hence, the test product does not produce clastogenic effects.

### 3.6. In Vivo Micronucleus Assay

LN19183 administration did not significantly alter the bone marrow micronucleated polychromatic erythrocyte (MNPCE) population in the experimental mice (Table 7). As expected, cyclophosphamide increased the MNPCE population in the treated mice compared to the untreated mice. The ratio of PCE/TE in the LN19183 or cyclophosphamide-treated mice is unaltered compared to the vehicle control–treated mice (Table 7).

## 4. Discussion

This investigation evaluates the comprehensive safety of a proprietary, synergistic botanical composition, LN19183, in OECD-approved in vitro and in vivo toxicity studies. LN19183 is a standardized, synergistic combination of *C. aurantifolia* fruit peel (CA) and *T. cacao* bean (TC) extracts [1]. Although individual plant materials are widely consumed and considered safe, according to regulatory authorities such as the USFDA or the European Food Safety Authority (EFSA), a comprehensive range safety profile of a new botanical composition is crucial [30]. The OECD-recommended preclinical toxicity studies are considered useful in evaluating the safety of a drug or chemical test substance in humans [31]. This investigation demonstrates the safety profile of LN19183 in short-term and long-term systemic and genetic toxicity studies.

The single-dose oral and dermal toxicity studies suggest that LN19183 treatment exhibits no toxicity, with oral median lethal doses (LD50) greater than 5000 and 2000 mg per kg BW, respectively. The calculated LD50 values suggest that the botanical composition is unclassified or in category 5, indicating no significant toxic effects [29]. Furthermore, LN19183 did not show gross toxicity or signs of dermal and eye irritation in white New Zealand rabbits. These observations conclude that LN19183 does not irritate the eyes or skin [17, 29].

The present subchronic toxicity study shows that daily oral dose of LN19183 up to 2000 mg per kg BW did not show clinical signs, changes in behavior, or growth in the experimental rats compared to the gender-specific controls. The main or the reversal group rats were healthy and survived till the end of the study. Although LN19183-fed male and female reversal rats consumed significantly reduced food on the 4th and 5th week, respectively, compared to the matched controls, their BWs did not alter significantly. The changes in food consumption were erratic and not considered a treatment-related effect. The LN19183-fed rats showed natural BW gain comparable to the control rats. Toxic chemical substances reduce food intake and adversely impact the normal growth of the treated animals [32]. Furthermore, LN19183 administration showed no changes in the rats' FOB. FOB helps assess whether supplementation is safe for the central nervous system (CNS) [33]. These observations indicate that LN19183 does not hamper normal growth or cause neurotoxicity in the rats.

Apart from a few exceptions, the hematology and plasma clinical biochemistry parameters of the LN19183-supplemented rats are unaltered compared to the untreated rats. However, these changes are within the normal physiological limits [34]. Abnormal clinical biochemistry findings are potential indicators of metabolic dysfunction and tissue damage due to the ingested product toxicity [35]. Unaltered lipid profiles and glucose levels indicate normal metabolism in the LN19183-supplemented rats. These observations are supported by no alteration in the rats' thyroids and parathyroid gland weights and the normal levels of serum thyroid hormones. These data indicate that LN19183 supplementation did not alter the thyroid function on lipid metabolism [17]. Furthermore, the serum liver transaminases, alkaline phosphatase, creatinine, and BUN supported by the macroscopic and microscopic observations on the liver and kidneys suggest that LN19183 supplementation did not cause toxicity on the normal structure and functions of these vital organs [36, 37]. Overall, the observations on BW, food intake, hematology and serum clinical biochemistry parameters, serum hormones, and pathology evaluations of the major organs suggest that LN19183 is not generally toxic to rats.

Furthermore, in the Ames assay, LN19183 did not produce revertant bacterial colonies, indicating any point mutation by substitution or frameshift in the bacterial strains' genome. Also, the micronucleus and chromosomal abnormality assay results consistently revealed that the LN19183 treatment did not induce structural abnormality or break in the chromosome. Together, the lack of genetic toxicity potential of LN19183 is a vital measure for this herbal blend's broad-spectrum safety.

## 5. Conclusion

These findings demonstrate that LN19183 does not cause general and genetic toxicity in the OECD-recommended in vitro and in vivo studies. The NOAEL for LN19183 in male and female rats is 2000 mg per kg BW/day in the 90-day oral toxicity study. This dose is equivalent to greater than 20 g/day for a human subject of 65 kg BW. These observations affirm the comprehensive safety of this novel botanical ingredient, thus suggesting that LN19183 consumption is safe for humans.

## Figures and Tables

**Table 1:** Effect of 90-day oral administration of LN19183 on hematology parameters of male and female Sprague Dawley rats.

Parameters	Sex	LN19183 dose (mg/kg body weight)					
		Main groups				Reversal groups	
		0	500	1000	2000	0	2000
WBC (10^9^ cells/L)	M	9.48 ± 2.07	8.78 ± 2.93	9.32 ± 1.91	7.89 ± 2.72	8.91 ± 1.74	9.01 ± 2.55
	F	6.13 ± 1.40	6.25 ± 1.39	6.05 ± 1.15	5.92 ± 1.26	6.00 ± 0.98	7.33 ± 2.60

Neutrophils (%)	M	19.98 ± 2.62	20.91 ± 2.62	20.19 ± 2.21	21.79 ± 2.36	21.56 ± 2.13	22.94 ± 2.00
	F	19.09 ± 1.72	22.24 ± 4.26	21.25 ± 2.15	21.16 ± 2.59	20.84 ± 2.31	19.44 ± 3.00

Lymphocytes (%)	M	77.22 ± 3.44	76.48 ± 3.02	76.96 ± 2.82	74.47 ± 3.65	74.78 ± 2.01	72.52 ± 1.78
	F	78.22 ± 2.48	75.01 ± 4.57	75.27 ± 2.89	74.71 ± 4.17	75.92 ± 4.00	78.40 ± 3.52

Monocytes (%)	M	2.04 ± 0.96	1.82 ± 0.80	1.74 ± 0.86	2.76 ± 1.75	2.66 ± 1.22	3.22 ± 1.13
	F	1.60 ± 0.89	1.74 ± 0.72	2.03 ± 1.42	2.95 ± 1.88	1.78 ± 1.29	1.02 ± 0.73

Eosinophils (%)	M	0.76 ± 0.34	0.79 ± 0.26	1.11 ± 0.50	0.98 ± 0.65	1.00 ± 0.26	1.32 ± 0.24
	F	1.09 ± 0.56	1.01 ± 0.42	1.45 ± 0.69	1.18 ± 0.75	1.46 ± 0.77	1.14 ± 0.34

Basophils (%)	M	0.00 ± 0.00	0.00 ± 0.00	0.00 ± 0.00	0.00 ± 0.00	0.00 ± 0.00	0.00 ± 0.00
	F	0.00 ± 0.00	0.00 ± 0.00	0.00 ± 0.00	0.00 ± 0.00	0.00 ± 0.00	0.00 ± 0.00

RBC (10^12^ cells/L)	M	9.32 ± 0.38	9.15 ± 0.54	9.47 ± 0.49	8.95 ± 0.62	9.40 ± 0.28	9.52 ± 0.43
	F	8.10 ± 0.38	8.48 ± 0.47	8.29 ± 0.33	8.28 ± 0.26	8.38 ± 0.21	8.45 ± 0.17

Hemoglobin (g/dL)	M	16.74 ± 0.44	16.89 ± 0.64	16.53 ± 0.64	16.57 ± 0.44	16.36 ± 0.26	16.18 ± 0.64
	F	15.33 ± 0.46	15.81 ± 0.59	15.74 ± 0.39	15.69 ± 0.37	15.42 ± 0.29	15.54 ± 0.51

Hematocrit (%)	M	48.17 ± 1.34	48.63 ± 1.85	48.27 ± 2.44	48.53 ± 1.54	47.72 ± 1.10	47.76 ± 1.75
	F	43.74 ± 1.91	45.70 ± 1.99	45.74 ± 1.57∗	45.92 ± 1.50∗	44.8 ± 0.65	45.58 ± 1.44

MCV (fL)	M	51.74 ± 1.80	53.25 ± 2.50	51.01 ± 1.75	54.36 ± 2.67∗	50.80 ± 1.13	50.24 ± 2.64
	F	54.05 ± 2.09	53.94 ± 1.55	55.26 ± 1.64	55.44 ± 1.30	53.5 ± 1.42	53.96 ± 2.22

MCH (pg)	M	17.98 ± 0.54	18.51 ± 0.82	17.46 ± 0.61	18.55 ± 0.98	17.44 ± 0.25	17.04 ± 0.99
	F	18.93 ± 0.73	18.67 ± 0.62	19.02 ± 0.63	18.99 ± 0.62	18.40 ± 0.47	18.38 ± 0.71

MCHC (g/dL)	M	34.76 ± 0.46	34.76 ± 0.59	34.24 ± 0.74	34.15 ± 0.53	34.30 ± 0.65	33.90 ± 0.48
	F	35.03 ± 0.67	34.62 ± 0.44	34.42 ± 0.63	34.22 ± 0.81∗	34.42 ± 0.36	34.06 ± 0.35

Platelets (10^9^ cells/L)	M	1036.0 ± 218.37	1094.7 ± 274.90	1157.4 ± 234.10	1049.2 ± 204.67	1004.20 ± 150.54	947.40 ± 125.43
	F	983.8 ± 139.36	1060.7 ± 355.37	1014.1 ± 208.97	1082.40 ± 247.65	869.6 ± 141.10	904.6 ± 242.45

Reticulocytes (%)	M	2.08 ± 0.33	2.20 ± 0.25	2.08 ± 0.33	2.07 ± 0.41	2.08 ± 0.30	2.24 ± 0.33
	F	2.26 ± 0.34	2.22 ± 0.32	2.14 ± 0.37	2.04 ± 0.35	2.08 ± 0.23	2.12 ± 0.41

Clotting time (sec)	M	126.00 ± 34.06	132.00 ± 35.21	132.00 ± 28.98	135.00 ± 29.15	96.00 ± 39.12	114.00 ± 39.12
	F	99.00 ± 28.46	120.00 ± 31.62	108.00 ± 25.30	117.00 ± 22.14	96.00 ± 39.12	102.00 ± 34.21

*Note:* Data are presented as mean ± SD. M and F indicate male and female rats. Each main group contains 20 rats (10 males and 10 females), and each reversal group contains 10 rats (5 males and 5 females).

Abbreviations: MCH, mean corpuscular hemoglobin; MCHC, mean corpuscular hemoglobin concentration; MCV, mean corpuscular volume; RBC, red blood cells; WBC, white blood cells.

^∗^Significant change (*p* < 0.05; one-way ANOVA) compared with the control group.

**Table 2:** Effect of 90-day oral administration of LN19183 on clinical biochemistry parameters of male and female Sprague Dawley rats.

Parameters	Sex	LN19183 dose (mg/kg body weight)					
		Main groups				Reversal groups	
		0	500	1000	2000	0	2000
Glucose (mg/dL)	M	123.6 ± 17.88	123.0 ± 20.67	124.70 ± 19.98	121.20 ± 12.07	119.8 ± 10.21	122.6 ± 9.56
	F	114.9 ± 11.01	112.9 ± 9.72	124 ± 10.76	125.3 ± 6.52	118.0 ± 5.52	120.0 ± 7.52

Blood urea nitrogen (mg/dL)	M	19.0 ± 3.59	18.50 ± 2.27	18.40 ± 1.9	18.10 ± 2.13	18.4 ± 2.61	19.8 ± 1.64
	F	18.7 ± 3.09	21.9 ± 2.77	17.6 ± 2.01	20 ± 4.67	19.4 ± 1.67	19.8 ± 2.28

Urea (mg/dL)	M	40.8 ± 7.63	39.5 ± 4.99	39.4 ± 3.86	38.70 ± 4.52	39.8 ± 5.81	42.2 ± 2.95
	F	40.2 ± 6.75	46.8 ± 5.67	37.9 ± 4.53	42.6 ± 9.97	41.8 ± 3.96	42.4 ± 4.83

Creatinine (mg/dL)	M	0.48 ± 0.08	0.46 ± 0.09	0.40 ± 0.10	0.41 ± 0.04	0.48 ± 0.09	0.39 ± 0.06
	F	0.48 ± 0.12	0.46 ± 0.07	0.45 ± 0.09	0.48 ± 0.12	0.54 ± 0.11	0.58 ± 0.16

Total cholesterol (mg/dL)	M	90.1 ± 23.52	98.4 ± 14.33	96.6 ± 17.98	90.8 ± 15.76	102.8 ± 17.85	99.8 ± 26.08
	F	99.7 ± 10.07	113.3 ± 16.68	110 ± 17.22	111.5 ± 17.32	108.0 ± 23.95	117.40 ± 7.40

Triglyceride (mg/dL)	M	83.9 ± 31.34	81.8 ± 15.16	90.5 ± 37.87	78.9 ± 28.38	72.6 ± 28.42	86 ± 33.65
	F	78.3 ± 22.09	97.6 ± 42.94	67.6 ± 28.2	85 ± 37.46	97.2 ± 13.74	84.0 ± 11.20

LDL (mg/dL)	M	21.7 ± 4.97	23.3 ± 3.53	21.3 ± 4.35	21.7 ± 4.0	25.4 ± 4.83	27 ± 7.68
	F	12.4 ± 1.51	15.7 ± 3.77	14.6 ± 3.6	15.7 ± 2.67	16.4 ± 5.68	16.8 ± 3.11

HDL (mg/dL)	M	40.1 ± 11.8	43.1 ± 6.52	45.4 ± 12.7	40.4 ± 6.93	63.4 ± 10.74	64.8 ± 21.73
	F	57.4 ± 5.62	64.1 ± 8.92	61.6 ± 6.36	64.6 ± 15.74	76.8 ± 16.28	86.2 ± 7.56

Total bilirubin (mg/dL)	M	0.11 ± 0.03	0.13 ± 0.05	0.12 ± 0.04	0.13 ± 0.05	0.12 ± 0.04	0.12 ± 0.04
	F	0.12 ± 0.04	0.12 ± 0.04	0.13 ± 0.05	0.11 ± 0.03	0.16 ± 0.05	0.12 ± 0.04

AST (U/L)	M	140.1 ± 25.05	135.8 ± 13.71	119.4 ± 18.41	141.0 ± 35.06	120.4 ± 7.64	111.6 ± 21.42
	F	135 ± 24.74	118.9 ± 17.43	117.9 ± 17.57	121 ± 25.43	123.2 ± 13.42	114.2 ± 4.82

ALT (U/L)	M	49.5 ± 4.79	51.8 ± 11.58	48.4 ± 7.66	53.0 ± 18.23	66 ± 16.72	57.4 ± 10.38
	F	42.25 ± 18.05	45.6 ± 10.41	45.1 ± 14.17	42.1 ± 5.84	55.8 ± 14.32	52.6 ± 6.84

Alkaline phosphatase (U/L)	M	163.5 ± 19.4	158.1 ± 22.74	141.8 ± 27.71	166.0 ± 30.27	147.8 ± 30.59	151.4 ± 37.64
	F	117.5 ± 30.16	128.5 ± 39.2	135 ± 44.27	150.2 ± 36.98	105.0 ± 35.14	103 ± 25.45

Total protein (g/dL)	M	7.04 ± 0.21	6.87 ± 0.28	6.84 ± 0.22	6.80 ± 0.20	7.08 ± 0.26	6.88 ± 0.22
	F	6.73 ± 0.36	6.71 ± 0.28	6.67 ± 0.22	6.59 ± 0.24	6.58 ± 0.13	6.74 ± 0.27

Albumin (g/dL)	M	3.8 ± 0.09	3.71 ± 0.11	3.7 ± 0.08	3.69 ± 0.12	3.76 ± 0.05	3.76 ± 0.11
	F	3.73 ± 0.2	3.71 ± 0.14	3.68 ± 0.15	3.71 ± 0.12	3.62 ± 0.16	3.72 ± 0.13

Calcium (mg/dL)	M	10.15 ± 0.3	10.25 ± 0.13	10.3 ± 0.33	9.79 ± 0.42	10.62 ± 0.24	10.5 ± 0.3
	F	10.43 ± 0.29	10.36 ± 0.19	10.28 ± 0.3	10.13 ± 0.35	10.42 ± 0.27	10.5 ± 0.27

Phosphorus (mg/dL)	M	6.24 ± 0.32	6.08 ± 0.42	6.18 ± 0.40	6.48 ± 0.96	6.18 ± 0.41	6.00 ± 0.58
	F	4.79 ± 0.58	5.13 ± 0.57	5.24 ± 0.45	5.27 ± 0.6	4.62 ± 1.08	5.24 ± 0.88

Sodium (mmoL/L)	M	138.37 ± 0.81	138.81 ± 1.05	138.66 ± 0.92	138.35 ± 0.81	138.2 ± 1.89	138.12 ± 0.27
	F	138.83 ± 1.25	138.24 ± 1.32	138.16 ± 1.11	138.07 ± 1.17	137.18 ± 1.17	137.84 ± 0.69

Potassium (mmoL/L)	M	4.64 ± 0.29	4.51 ± 0.35	4.72 ± 0.24	4.56 ± 0.24	5.08 ± 0.37	4.97 ± 0.31
	F	4.33 ± 0.3	4.38 ± 0.31	4.28 ± 0.32	4.42 ± 0.31	4.61 ± 0.33	4.31 ± 0.3

Chloride (mmoL/L)	M	99.68 ± 0.87	100.01 ± 1.13	98.95 ± 1.4	98.58 ± 1.02	100.4 ± 1.77	99.26 ± 0.8
	F	99.67 ± 1.45	99.16 ± 1.23	99.17 ± 0.96	99.73 ± 1.34	100.92 ± 0.76	100.6 ± 2.02

*Note:* Data are presented as mean ± SD. M and F indicate male and female rats. Each main group contains 20 rats (10 males and 10 females), and each reversal group contains 10 rats (5 males and 5 females).

Abbreviations: ALT, alanine aminotransferase; AST, aspartate aminotransferase; BUN, blood urea nitrogen; HDL, high-density lipoprotein; LDL, low-density lipoprotein.

**Table 3:** Effect of 90-day oral administration of LN19183 on the body weights of male and female Sprague Dawley rats.

Weeks of treatment	Sex	LN19183 dose (mg/kg BW)					
		Main groups				Reversal groups	
		0	500	1000	2000	0	2000
0	M	252.67 ± 8.54	254.77 ± 11.3	257.15 ± 14.00	256.21 ± 10.23	253.12 ± 9.86	254.79 ± 9.56
	F	183.29 ± 10.93	184.67 ± 10.62	187.81 ± 14.65	185.77 ± 13.61	184.67 ± 9.19	184.63 ± 12.4

1	M	284.39 ± 16.34	292.01 ± 16.70	292.76 ± 14.82	286.53 ± 11.72	282.51 ± 10.39	287.71 ± 17.95
	F	199.35 ± 11.82	203.06 ± 10.65	201.94 ± 16.77	205.08 ± 11.36	199.44 ± 4.57	199.44 ± 10.4

2	M	310.58 ± 21.28	322.25 ± 24.28	318.00 ± 17.09	309.85 ± 19.37	318.41 ± 23.30	311.28 ± 24.53
	F	212.24 ± 13.66	214.68 ± 10.08	213.07 ± 16.52	214.41 ± 14.01	213.27 ± 7.9	212.35 ± 9.18

3	M	331.91 ± 24.14	345.19 ± 29.85	339.15 ± 20.86	330.46 ± 22.63	340.61 ± 31.21	331.04 ± 31.68
	F	221.88 ± 17.68	221.83 ± 10.57	224.18 ± 19.13	226.71 ± 17.60	223.53 ± 7.53	221.9 ± 14.49

4	M	353.99 ± 25.41	365.27 ± 33.51	360.94 ± 20.68	350.30 ± 28.62	362.87 ± 37.02	352.64 ± 37.07
	F	230.78 ± 20.79	231.29 ± 12.3	234.91 ± 23.69	233.71 ± 17.94	229.76 ± 8.21	229.26 ± 13.35

5	M	368.42 ± 28.88	379.73 ± 35.26	377.71 ± 20.75	362.79 ± 30.89	377.46 ± 42.06	370.61 ± 40.44
	F	234.66 ± 20.88	236.71 ± 11.73	242.3 ± 25.35	239.16 ± 17.87	241.08 ± 12.35	232.42 ± 13.33

6	M	384.83 ± 30.94	400.36 ± 40.15	394.44 ± 23.33	377.96 ± 33.55	395.84 ± 44.08	385.59 ± 42.95
	F	245.09 ± 20.61	246.52 ± 16.20	246.55 ± 24.61	247.08 ± 18.38	247.56 ± 11.84	238.04 ± 11.00

7	M	395.83 ± 30.74	412.54 ± 41.66	409.35 ± 25.93	390.57 ± 36.04	412.20 ± 48.10	399.60 ± 48.10
	F	250.84 ± 24.23	251.98 ± 17.07	252.00 ± 23.46	252.47 ± 17.91	253.96 ± 9.6	243.88 ± 12.24

8	M	406.09 ± 31.44	424.95 ± 42.14	417.77 ± 24.56	399.53 ± 36.70	417.05 ± 44.87	407.53 ± 49.74
	F	254.51 ± 23.4	255.96 ± 19.24	255.69 ± 22.97	256.02 ± 19.61	256.5 ± 10.52	247.52 ± 12.87

9	M	420.30 ± 32.99	440.91 ± 47.09	432.47 ± 25.90	414.28 ± 40.71	431.81 ± 45.82	414.33 ± 50.69
	F	260.00 ± 22.23	261.18 ± 22.50	260.11 ± 23.31	261.32 ± 18.91	260.89 ± 11.19	251.55 ± 10.63

10	M	432.45 ± 36.05	448.78 ± 50.09	445.44 ± 26.26	423.72 ± 43.02	442.37 ± 49.84	426.70 ± 53.72
	F	265.5 ± 24.82	264.36 ± 21.11	266.15 ± 22.57	265.77 ± 20.02	267.27 ± 11.55	257.75 ± 13.41

11	M	439.65 ± 34.32	456.18 ± 52.68	450.64 ± 27.28	430.47 ± 42.98	447.35 ± 47.02	430.52 ± 55.32
	F	269.49 ± 26.47	267.64 ± 20.58	269.96 ± 23.7	271.59 ± 21.21	271.2 ± 10.26	258.77 ± 13.23

12	M	445.11 ± 35.08	462.72 ± 55.52	461.18 ± 31.06	437.95 ± 43.41	455.08 ± 50.50	437.43 ± 56.56
	F	271.66 ± 26.59	271.16 ± 20.65	273.98 ± 24.81	274.37 ± 20.68	272.62 ± 10.74	262.06 ± 12.41

13	M	448.27 ± 35.45	467.01 ± 56.55	464.79 ± 32.16	443.16 ± 43.12	459.61 ± 51.49	445.99 ± 59.88
	F	267.83 ± 17.67	273.68 ± 22.17	277.95 ± 26.53	278.76 ± 21.18	278.22 ± 10.91	268.12 ± 12.26

14	M	—	—	—	—	468.96 ± 48.35	453.48 ± 57.17
	F	—	—	—	—	281.22 ± 9.74	270.31 ± 12.69

15	M	—	—	—	—	476.72 ± 49.91	461.82 ± 58.78
	F	—	—	—	—	283.82 ± 10.46	273.20 ± 11.44

16	M	—	—	—	—	484.08 ± 50.31	469.13 ± 61.15
	F	—	—	—	—	286.56 ± 8.78	277.80 ± 13.52

17	M	—	—	—	—	486.20 ± 50.83	470.72 ± 61.91
	F					290.21 ± 5.68	284.52 ± 11.01

*Note:* Data are presented as mean ± SD of body weight (BW) in g. M and F indicate male and female rats. Each main group contains 20 rats (10 males and 10 females), and each reversal group contains 10 rats (5 males and 5 females). Week 0 indicates before starting the treatment; week 13 and 17 data present fasting body weights.

**Table 4:** Effect of 90-day oral administration of LN19183 on food consumption by male and female Sprague Dawley rats.

Weeks of treatment	Sex	LN19183 dose (mg/kg BW)					
		Main groups				Reversal groups	
		0	500	1000	2000	0	2000
1	M	153.09 ± 9.36	164.49 ± 4.40∗	158.27 ± 9.26	156.48 ± 10.93	168.69 ± 2.49	156.03 ± 15.13
	F	106.16 ± 6.90	114.78 ± 5.44∗	103.63 ± 8.47	110.67 ± 5.72	105.96 ± 0.10	106.81 ± 2.87

2	M	162.20 ± 9.71	164.79 ± 5.12	159.49 ± 6.57	154.43 ± 10.13	166.42 ± 0.10	154.45 ± 11.94
	F	115.13 ± 7.05	112.13 ± 3.58	107.38 ± 5.95	109.85 ± 6.92	114.09 ± 0.92	112.60 ± 2.25

3	M	153.31 ± 8.51	157.78 ± 3.74	154.82 ± 6.09	149.64 ± 10.24	158.83 ± 0.86	151.58 ± 14.26
	F	108.32 ± 3.40	103.06 ± 4.86	103.02 ± 8.90	107.47 ± 8.92	107.58 ± 2.96	108.89 ± 3.30

4	M	160.19 ± 9.91	164.70 ± 5.01	162.26 ± 4.93	156.26 ± 11.20	166.95 ± 4.27	158.34 ± 8.65∗
	F	112.32 ± 3.93	115.71 ± 9.88	111.03 ± 12.54	110.26 ± 5.28	111.79 ± 4.18	112.44 ± 12.80

5	M	159.04 ± 7.35	159.55 ± 5.04	158.60 ± 7.24	152.34 ± 9.01	164.38 ± 0.33	154.49 ± 11.89
	F	109.35 ± 6.11	110.46 ± 7.05	109.76 ± 12.93	109.52 ± 4.76	109.12 ± 4.66	103.83 ± 5.18∗

6	M	158.29 ± 8.24	160.22 ± 4.23	159.09 ± 7.89	155.64 ± 8.88	164.69 ± 0.99	153.90 ± 11.89
	F	111.57 ± 7.39	113.60 ± 8.12	104.12 ± 5.83	113.21 ± 7.15	105.88 ± 3.76	107.39 ± 2.83

7	M	152.06 ± 11.10	153.89 ± 3.79	154.22 ± 8.54	148.53 ± 10.77	159.72 ± 0.67	150.40 ± 13.16
	F	106.61 ± 7.05	106.23 ± 7.35	99.45 ± 4.85	108.44 ± 6.59	102.08 ± 0.86	102.81 ± 1.82

8	M	160.05 ± 10.62	160.32 ± 3.65	154.08 ± 7.79	151.58 ± 5.78	156.12 ± 5.44	151.42 ± 6.57
	F	108.76 ± 10.96	115.43 ± 8.27	103.10 ± 9.03	109.11 ± 7.75	105.75 ± 2.39	103.95 ± 7.13

9	M	159.71 ± 9.86	161.10 ± 5.36	155.60 ± 8.73	151.90 ± 10.31	164.16 ± 0.12	161.25 ± 7.08
	F	104.90 ± 8.51	111.68 ± 10.64	112.27 ± 22.89	109.09 ± 7.46	102.31 ± 3.00	104.07 ± 0.49

10	M	155.71 ± 10.83	157.34 ± 5.38	155.01 ± 8.71	151.33 ± 7.58	159.34 ± 2.83	161.41 ± 4.09
	F	106.83 ± 3.38	103.56 ± 4.81	104.83 ± 8.72	108.34 ± 7.25	105.20 ± 0.26	105.06 ± 0.91

11	M	151.95 ± 8.05	154.13 ± 4.76	153.11 ± 10.29	146.96 ± 7.80	154.82 ± 2.93	156.05 ± 0.46
	F	106.16 ± 8.31	104.60 ± 5.21	100.76 ± 9.69	106.66 ± 7.23	104.21 ± 3.59	106.87 ± 4.48

12	M	154.52 ± 6.89	157.02 ± 6.74	156.70 ± 13.67	150.82 ± 4.38	156.00 ± 2.76	149.35 ± 6.35
	F	102.04 ± 6.10	107.72 ± 8.24	105.88 ± 4.75	110.84 ± 7.69∗	101.33 ± 0.49	104.80 ± 4.53

13	M	109.46 ± 4.44	109.11 ± 7.06	109.95 ± 8.30	118.42 ± 15.10	153.43 ± 2.62	151.09 ± 1.20
	F	70.63 ± 9.72	75.05 ± 8.16	70.76 ± 6.29	75.80 ± 1.20	107.91 ± 7.95	106.69 ± 0.00

14	M	—	—	—	—	152.88 ± 3.22	145.36 ± 6.71
	F	—	—	—	—	103.18 ± 1.61	109.10 ± 7.97

15	M	—	—	—	—	160.13 ± 2.07	157.84 ± 12.71
	F	—	—	—	—	98.92 ± 2.07	106.55 ± 6.88

16	M	—	—	—	—	157.99 ± 0.12	155.48 ± 8.62
	F	—	—	—	—	97.71 ± 2.35	107.36 ± 7.46∗

17	M	—	—	—	—	110.89 ± 2.70	113.88 ± 6.61
	F	—	—	—	—	78.80 ± 3.64	79.11 ± 5.96

*Note:* Data are presented as mean ± SD of body weight (BW) in g. M and F indicate male and female rats. Each main group contains 20 rats (10 males and 10 females), and each reversal group contains 10 rats (5 males and 5 females). The animals were once fasted overnight for blood sampling on weeks 13 and 17.

^∗^Significant changes (*p* < 0.05; one-way ANOVA) compared with the control groups.

**Table 5:** Effect of LN19183 treatment on bacterial reverse mutation in the presence or absence of the metabolic activation.

Treatments	μg/plate	*S. typhimurium* strains				*E. coli*
		TA98	TA100	TA1535	TA1537	WP2 uvrA
*Revertant colonies/plate (mean ± SD) in the presence of S9*						
DMSO	0.0	23.33 ± 2.08	109.67 ± 5.86	13.67 ± 1.53	9.00 ± 1.00	10.00 ± 2
LN19183	312.5	23.00 ± 1.00	103.67 ± 5.13	9.67 ± 1.15	10.33 ± 3.06	13.33 ± 1.15
	625	21.00 ± 2.65	98.00 ± 9.54	13.67 ± 3.21	7.33 ± 1.15	13.00 ± 1.00
	1250	22.33 ± 4.51	96.00 ± 5.29	8.67 ± 3.21	7.33 ± 0.58	9.67 ± 2.08
	2500	24.33 ± 5.13	102.33 ± 3.06	12.33 ± 3.51	9.67 ± 1.53	14.00 ± 2.65
	5000	22.33 ± 2.52	98.33 ± 2.08	10.00 ± 1.73	10.67 ± 2.08	13.67 ± 3.21
2-Aminoanthracene	20	1192.67 ± 140.12∗	969.33 ± 104.33∗	649.33 ± 58.01∗	151.67 ± 19.5∗	—
2-Aminoanthracene	30	—	—	—	—	169.00 ± 18.68∗

*Revertant colonies/plate (mean ± SD) in the absence of S9*						
DMSO	0	24.00 ± 2.65	100.33 ± 8.08	15.00 ± 1.73	12.00 ± 2.65	9.67 ± 2.08
LN19183	312.5	24.67 ± 4.04	106.33 ± 9.29	13.33 ± 4.51	9.67 ± 3.51	11.00 ± 1.00
	625	23.67 ± 3.79	99.67 ± 8.14	12.33 ± 3.06	8.00 ± 3.61	10.00 ± 3.46
	1250	22.33 ± 4.51	103.00 ± 3.46	12.67 ± 3.06	9.33 ± 2.52	10.33 ± 3.51
	2500	22.33 ± 2.52	102.67 ± 7.23	11.67 ± 3.06	8.00 ± 2.00	12.33 ± 2.08
	5000	23.33 ± 4.16	99.67 ± 6.66	11.67 ± 3.51	8.67 ± 0.58	9.67 ± 1.15
2-Nitrofluorene	25	1173.33 ± 88.12∗	—	—	—	—
Sodium azide	10	—	1309.33 ± 59.8∗	953.33 ± 84.88∗	—	—
9-Aminoacridine	50	—	—	—	348.67 ± 27.30∗	—
4-Nitroquinoline	3	—	—	—	—	233.33 ± 36.02∗

Abbreviation: DMSO, dimethyl sulfoxide.

^∗^Significant change (*p* < 0.05) compared to the DMSO or vehicle control culture using one-way ANOVA.

**Table 6:** Frequency of micronucleated cells in the LN19183-treated human peripheral blood lymphocytes in vitro.

Treatments	Conc. (μg/mL)	No. of cells counted	Average of micronucleated cells		
			−S9		+S9
			4 h	24 h	4 h
DMSO	0	1000	2.50	1.50	2.50

LN19183	312.5	1000	1.50	1.50	1.50
	625	1000	2.00	2.00	1.50
	1250	1000	2.00	1.50	1.00

Mitomycin C	0.3	1000	20.00	17.00∗	—

Colchicine	0.03	1000	19.50∗	—	—
	0.01	1000	—	16.50∗	—

Cyclophosphamide	10	1000	—	—	17.50∗

Abbreviation: DMSO, dimethyl sulfoxide.

^∗^Significant change (*p* < 0.05) compared to the DMSO or vehicle control culture using one-way ANOVA.

**Table 7:** Micronucleated polychromatic erythrocytes in the bone marrow samples of LN19183-treated mice.

Treatment group	Dose (mg/kg BW)	Male (*n* = 5)		Female (*n* = 5)	
		PCE:TE	% MNPCE	PCE:TE	% MNPCE
Vehicle control (distilled water)	0	0.557	0.04	0.547	0.02

LN19183	500	0.537	0.03	0.530	0.03
	1000	0.531	0.03	0.523	0.02
	2000	0.530	0.04	0.533	0.03

Cyclophosphamide	40	0.527	1.28∗	0.522	1.13∗

Abbreviations: BW, body weight; MNPCE, micronucleated polychromatic erythrocyte; PCE, polychromatic erythrocyte; TE, total erythrocytes.

^∗^Significant change (*p* < 0.05) compared with the vehicle control using one-way ANOVA.

## Data Availability

The data obtained from the experiments are presented in the article; any additional data will be available upon request.

## Supporting Information

Table S1: mortality and gross pathological observations in acute oral study. Table S2: serum hormone levels in male and female Sprague Dawley rats. Table S3: absolute and relative organ weight of male Sprague Dawley rats. Table S4: absolute and relative organ weight of female Sprague Dawley rats. Table S5: frequency of chromosome aberrations in LN19183-treated human peripheral blood lymphocytes in vitro.
