# Supplementary material for: Acute, Subchronic, and Genetic Toxicity Assessments of a Composition of Citrus aurantifolia Fruit Rind and Theobroma cacao Seed Extracts
Source: J Toxicol. 2024 Nov 27;2024:4239607. doi: 10.1155/jt/4239607 (PMC11617045; doi:10.1155/jt/4239607)
Supplement: Supporting Information — Additional supporting information can be found online in the Supporting Information section. [file 4239607.f1.docx]

**Supplementary Table S1: Mortality and gross pathological observations in acute oral study**

| Observations | Animal Number | | |
| --- | --- | --- | --- |
|  | 1 | 2 | 3 |
| Mortality | No | No | No |
| Clinical Signs | No | No | No |
| Behavioral changes | No | No | No |
| Distress | No | No | No |
| Body Weight (in g.) | Initial:166.89 | Initial:164.20 | Initial:162.60 |
|  | Final:189.56 | Final:186.63 | Final:183.12 |
| Gross Pathology | NAD | NAD | NAD |
| Days of observation | 14 | 14 | 14 |

NAD: No abnormality detected. Clinical signs include piloerection, hunched posture, loose feces, gastroesophageal reflux, salivation, muscle tremors, weakness.

**Supplementary Table S2. Effect of 90-day oral administration of LN19183 on serum hormone levels in male and female Sprague Dawley rats.**

| Measurements | Sex | LN19183 Dose (mg/kg BW) | | | | | |
| --- | --- | --- | --- | --- | --- | --- | --- |
|  |  | Main Groups | | | | Reversal Groups | |
|  |  | 0 | 500 | 1000 | 2000 | 0 | 2000 |
| T3 (ng/mL) | M | 0.37 ± 0.04 | 0.38 ± 0.04 | 0.37 ± 0.02 | 0.34 ± 0.02 | 0.28 ± 0.02 | 0.28 ± 0.04 |
|  | F | 0.36 ± 0.03 | 0.37 ± 0.03 | 0.33 ± 0.03 | 0.35 ± 0.04 | 0.25 ± 0.04 | 0.27 ± 0.03 |
| T4 ( ng/ml ) | M | 23.06 ± 2.09 | 19.21 ± 1.71 | 19.87 ± 3.19 | 20.19 ± 1.80 | 17.18 ± 7.54 | 13.82 ± 1.98 |
|  | F | 20.99 ± 1.87 | 18.87 ± 1.55 | 19.84 ± 2.48 | 21.62 ± 3.88 | 18.37 ± 6.14 | 16.54 ± 5.46 |
| TSH (ng/mL) | M | 0.50 ± 0.05 | 0.46 ± 0.03 | 0.46 ± 0.06 | 0.46 ± 0.05 | 0.56 ± 0.04 | 0.52 ± 0.03 |
|  | F | 0.47 ± 0.03 | 0.46 ± 0.03 | 0.45 ± 0.06 | 0.46 ± 0.05 | 0.52 ± 0.05 | 0.51 ± 0.04 |

Data are presented as mean ± SD. M and F indicate male and female rats. T3, Triiodothyronine; T4, Thyroxine; TSH, Thyroid Stimulating Hormone. Each main group contains 20 rats (10 male and 10 female), and each reversal group contains 10 rats (5 male and 5 female).

**Supplementary Table S3: Effect of 90-day oral administration of LN19183 on absolute and relative organ weight of male Sprague Dawley rats.**

| Organs | LN19183 Dose (mg/kg BW) | | | | | |
| --- | --- | --- | --- | --- | --- | --- |
|  | Main Groups | | | | Reversal Groups | |
|  | 0 | 500 | 1000 | 2000 | 0 | 2000 |
| Liver | 15.44 ± 1.73 | 15.73 ± 1.95 | 16.34 ± 2.11 | 15.99 ± 2.48 | 14.43 ± 1.52 | 14.91 ± 3.91 |
| % of BW | 3.59 ± 0.30 | 3.50 ± 0.22 | 3.64 ± 0.35 | 3.73 ± 0.44 | 3.06 ± 0.28 | 3.22 ± 0.47 |
| Kidneys | 3.36 ± 0.35 | 3.32 ± 0.59 | 3.39 ± 0.48 | 3.12 ± 0.30 | 3.26 ± 0.01 | 3.15 ± 0.38 |
| % of BW | 0.78 ± 0.07 | 0.74 ± 0.10 | 0.75 ± 0.09 | 0.73 ± 0.08 | 0.69 ± 0.07 | 0.69 ± 0.06 |
| Adrenal Glands | 0.07 ± 0.01 | 0.08 ± 0.02 | 0.07 ± 0.01 | 0.07 ± 0.01 | 0.07 ± 0.01 | 0.08 ± 0.00 |
| % of BW | 0.02 ± 0.00 | 0.02 ± 0.00 | 0.02 ± 0.00 | 0.02 ± 0.00 | 0.02 ± 0.00 | 0.02 ± 0.00 |
| Heart | 1.54 ± 0.16 | 1.60 ± 0.17 | 1.63 ± 0.12 | 1.50 ± 0.17 | 1.46 ± 0.10 | 1.46 ± 0.18 |
| % of BW | 0.36 ± 0.02 | 0.36 ± 0.02 | 0.36 ± 0.02 | 0.35 ± 0.03 | 0.31 ± 0.02 | 0.32 ± 0.03 |
| Brain | 2.28 ± 0.17 | 2.29 ± 0.16 | 2.13 ± 0.13 | 2.13 ± 0.11 | 2.14 ± 0.09 | 2.10 ± 0.07 |
| % of BW | 0.53 ± 0.04 | 0.52 ± 0.06 | 0.48 ± 0.04 | 0.50 ± 0.06 | 0.46 ± 0.05 | 0.47 ± 0.06 |
| Spleen | 0.89 ± 0.12 | 0.88 ± 0.12 | 0.89 ± 0.12 | 0.82 ± 0.14 | 0.80 ± 0.06 | 0.81 ± 0.14 |
| % of BW | 0.21 ± 0.02 | 0.20 ± 0.03 | 0.20 ± 0.02 | 0.19 ± 0.03 | 0.17 ± 0.02 | 0.18 ± 0.02 |
| Thymus | 0.52 ± 0.19 | 0.51 ± 0.10 | 0.49 ± 0.11 | 0.46 ± 0.07 | 0.36 ± 0.11 | 0.38 ± 0.10 |
| % of BW | 0.12 ± 0.04 | 0.11 ± 0.02 | 0.11 ± 0.02 | 0.11 ± 0.01 | 0.08 ± 0.02 | 0.08 ± 0.01 |
| Testes | 3.68 ± 0.19 | 3.80 ± 0.49 | 3.75 ± 0.37 | 3.67 ± 0.33 | 3.5 ± 0.16 | 3.44 ± 0.31 |
| % of BW | 0.86 ± 0.09 | 0.85 ± 0.10 | 0.84 ± 0.01 | 0.86 ± 0.10 | 0.75 ± 0.07 | 0.77 ± 0.16 |
| Epididymides | 1.54 ± 0.12 | 1.56 ± 0.24 | 1.48 ± 0.21 | 1.47 ± 0.17 | 1.50 ± 0.19 | 1.39 ± 0.09 |
| % of BW | 0.36 ± 0.05 | 0.35 ± 0.08 | 0.33 ± 0.05 | 0.34 ± 0.04 | 0.32 ± 0.03 | 0.31 ± 0.05 |
| SV-CG and Prostate Gland | 3.30 ± 0.44 | 3.16 ± 0.26 | 3.21 ± 0.31 | 3.16 ± 0.14 | 3.26 ± 0.71 | 3.43 ± 0.33 |
| % of BW | 0.77 ± 0.12 | 0.71 ± 0.12 | 0.72 ± 0.11 | 0.74 ± 0.07 | 0.70 ± 0.21 | 0.76 ± 0.15 |
| Thyroid with parathyroid | 0.02 ± 0.00 | 0.02 ± 0.00 | 0.02 ± 0.00 | 0.03 ± 0.00 | 0.03 ± 0.01 | 0.03 ± 0.00 |
| % of BW | 0.01 ± 0.00 | 0.01 ± 0.00 | 0.01 ± 0.00 | 0.01 ± 0.00 | 0.01 ± 0.00 | 0.01 ± 0.00 |
| Pituitary Gland | 0.01 ± 0.00 | 0.01 ± 0.00 | 0.01 ± 0.00 | 0.01 ± 0.00 | 0.01 ± 0.00 | 0.01 ± 0.00 |
| % of BW | 0.00 ± 0.00 | 0.00 ± 0.00 | 0.00 ± 0.00 | 0.00 ± 0.00 | 0.00 ± 0.00 | 0.00 ± 0.00 |

Data are presented as mean ± SD. The organ weights are in grams. Each main and reversal group contains 10 and 5 male rats, respectively. BW, body weight; SV-CG, seminal vesicles with coagulating glands.

**Supplementary Table S4: Effect of 90-day oral administration of LN19183 on absolute and relative organ weight of female Sprague Dawley rats.**

| Organs | LN19183 Dose (mg/kg BW) | | | | | |
| --- | --- | --- | --- | --- | --- | --- |
|  | Main Groups | | | | Reversal Groups | |
|  | 0 | 500 | 1000 | 2000 | 0 | 2000 |
| Liver | 8.40 ± 0.84 | 8.43 ± 0.90 | 9.08 ± 0.89 | 9.33 ± 0.82 | 7.52 ± 1.04 | 8.05 ± 1.2 |
| % of BW | 3.23 ± 0.31 | 3.23 ± 0.23 | 3.42 ± 0.30 | 3.51 ± 0.23 | 2.68 ± 0.32 | 2.96 ± 0.39 |
| Kidneys | 1.81 ± 0.14 | 1.84 ± 0.24 | 2.02 ± 0.34 | 1.84 ± 0.19 | 1.80 ± 0.20 | 1.67 ± 0.17 |
| % of BW | 0.70 ± 0.05 | 0.70 ± 0.07 | 0.76 ± 0.11 | 0.69 ± 0.07 | 0.64 ± 0.07 | 0.62 ± 0.05 |
| Adrenal Glands | 0.08 ± 0.01 | 0.08 ± 0.02 | 0.09 ± 0.02 | 0.08 ± 0.01 | 0.07 ± 0.01 | 0.08 ± 0.01 |
| % of BW | 0.03 ± 0.00 | 0.03 ± 0.01 | 0.04 ± 0.01 | 0.03 ± 0.00 | 0.03 ± 0.00 | 0.03 ± 0.00 |
| Heart | 0.99 ± 0.07 | 0.99 ± 0.09 | 1.02 ± 0.12 | 1.05 ± 0.12 | 0.93 ± 0.04 | 0.91 ± 0.05 |
| % of BW | 0.38 ± 0.03 | 0.38 ± 0.02 | 0.39 ± 0.03 | 0.39 ± 0.03 | 0.33 ± 0.01 | 0.34 ± 0.03 |
| Brain | 2.05 ± 0.11 | 1.98 ± 0.18 | 2.03 ± 0.16 | 2.01 ± 0.30 | 2.00 ± 0.10 | 2.04 ± 0.10 |
| % of BW | 0.79 ± 0.07 | 0.76 ± 0.05 | 0.77 ± 0.09 | 0.76 ± 0.14 | 0.71 ± 0.04 | 0.75 ± 0.03 |
| Spleen | 0.57 ± 0.19 | 0.65 ± 0.11 | 0.68 ± 0.10 | 0.65 ± 0.05 | 0.57 ± 0.04 | 0.62 ± 0.07 |
| % of BW | 0.22 ± 0.07 | 0.25 ± 0.03 | 0.26 ± 0.02 | 0.25 ± 0.02 | 0.20 ± 0.01 | 0.23 ± 0.02 |
| Thymus | 0.38 ± 0.08 | 0.43 ± 0.18 | 0.41 ± 0.12 | 0.35 ± 0.06 | 0.33 ± 0.03 | 0.30 ± 0.01 |
| % of BW | 0.14 ± 0.03 | 0.16 ± 0.07 | 0.15 ± 0.04 | 0.13 ± 0.02 | 0.12 ± 0.01 | 0.11 ± 0.02 |
| Uterus with Cervix | 0.69 ± 0.20 | 0.67 ± 0.21 | 0.67 ± 0.16 | 0.69 ± 0.13 | 0.62 ± 0.08 | 0.65 ± 0.12 |
| % of BW | 0.27 ± 0.08 | 0.25 ± 0.06 | 0.26 ± 0.07 | 0.26 ± 0.05 | 0.22 ± 0.03 | 0.24 ± 0.04 |
| Ovaries | 0.18 ± 0.03 | 0.21 ± 0.09 | 0.23 ± 0.05 | 0.17 ± 0.03 | 0.17 ± 0.03 | 0.20 ± 0.02 |
| % of BW | 0.07 ± 0.01 | 0.08 ± 0.03 | 0.09 ± 0.02 | 0.06 ± 0.01 | 0.06 ± 0.01 | 0.07 ± 0.01 |
| Thyroid with Parathyroid | 0.02 ± 0.00 | 0.03 ± 0.00 | 0.03 ± 0.00 | 0.02 ± 0.00 | 0.03 ± 0.01 | 0.029 ± 0.01 |
| % of BW | 0.01 ± 0.00 | 0.01 ± 0.00 | 0.01 ± 0.00 | 0.01 ± 0.00 | 0.01 ± 0.00 | 0.01 ± 0.00 |
| Pituitary Gland | 0.01 ± 0.00 | 0.02 ± 0.00 | 0.01 ± 0.00 | 0.016 ± 0.00 | 0.05 ± 0.08 | 0.02 ± 0.00 |
| % of BW | 0.00 ± 0.00 | 0.00 ± 0.00 | 0.00 ± 0.00 | 0.00 ± 0.00 | 0.00 ± 0.03 | 0.00 ± 0.00 |

Data are presented as mean ± SD. The organ weights are in grams. Each main and reversal group contains 10 and 5 female rats, respectively. BW, body weight.

**Supplementary Table S5: Frequency of chromosome aberrations in LN19183-treated human peripheral blood lymphocytes in vitro**

| Culture condition | Treatments | Conc. (µg/ml) | Mean mitotic Index | No. of cells Scored | % of chromosome aberrant cells |
| --- | --- | --- | --- | --- | --- |
| Short exposure (4 h) without S9 fraction | DMSO | 0 | 9.45 | 1005 | 0 |
|  | LN19183 | 312.5 | 7.75 | 1007 | 0 |
|  |  | 625 | 7.08 | 1003 | 0 |
|  |  | 1250 | 6.31 | 1014 | 0 |
|  | Mitomycin C | 0.3 | 6.78 | 1003 | 0 |
| Short exposure (4 h) with S9 fraction | DMSO | 0 | 9.05 | 1005 | 0 |
|  | LN19183 | 312.5 | 7.42 | 1011 | 0 |
|  |  | 625 | 6.38 | 1003 | 0 |
|  |  | 1250 | 5.79 | 1002 | 0 |
|  | Cyclophosphamide | 10 | 6.88 | 1003 | 0 |
| Long exposure (22 h) without S9 fraction | DMSO | 0 | 8.78 | 1014 | 0 |
|  | LN19183 | 312.5 | 7.02 | 1012 | 0 |
|  |  | 625 | 6.44 | 1009 | 0 |
|  |  | 1250 | 5.78 | 1004 | 0 |
|  | Mitomycin C | 0.3 | 6.40 | 1015 | 0 |

DMSO: Dimethyl sulfoxide
